# Co‐expression pattern of SLC transporter genes associated with the immune landscape and clinical outcomes in gastric cancer

**DOI:** 10.1111/jcmm.18003

**Published:** 2023-11-01

**Authors:** Yue Zhang, Zhihong Liu, Lingbo Li, Dongqiang Zeng, Huiying Sun, Jianhua Wu, Rui Zhou, Wangjun Liao

**Affiliations:** ^1^ Department of Oncology, Nanfang Hospital Southern Medical University Guangzhou China

**Keywords:** chemotherapy, gastric cancer, molecular subtype, prognosis, solute carrier transporter

## Abstract

Solute carrier (SLC) transporters play a dual role in the occurrence and progression of tumours by acting as both suppressors and promoters. However, the overall impact of SLC transcriptome signatures on the tumour microenvironment, biological behaviour and clinical stratification of gastric cancer has not been thoroughly investigated. Therefore, we comprehensively analysed the expression profiles of the SLC transporter family members to identify novel molecular subtypes in gastric cancer. We identified two distinct SLC subtypes, SLC‐S1 and SLC‐S2, using non‐negative matrix factorization. These subtypes were markedly linked with the tumour microenvironment landscape, biological pathway activation and distinct clinical features of gastric cancer. Furthermore, a new scoring model, the SLC score, was developed to quantify the SLC subtypes. High SLC scores indicated a pattern of ‘SLC‐S2’, characterized by stromal infiltration and activation, poor prognosis and insensitivity to chemotherapy and immunotherapy, but high sensitivity to imatinib. The SLC score could serve as a supplement to the Tumour Node Metastasis (TNM) staging system to guide personalized treatment strategies and predict prognosis for patients with gastric cancer.

## 1 INTRODUCTION

The solute carrier (SLC) transporter family is the second largest family of membrane proteins, comprising 456 known human SLC transporters.^1^ These transporters maintain cellular metabolic homeostasis by mediating the influx and efflux of diverse solutes, such as ions, nucleotides and sugars, across biofilms.^2^, ^3^ Previous studies revealed that SLC transporters are strongly linked to all reported cancer features, including tumour invasion, metastasis and drug resistance.^1^, ^4^ SLC transporters play dual roles in cancer development, and their potential to either suppress or promote tumours depends on their origin, epigenetic modifications and activation of related signalling pathways in different cancer types.^3^ For instance, SLC26A9 overexpression inhibits Wnt signalling to prevent gastric cancer development,^5^ whereas SLC34A2 overexpression promotes tumour growth and proliferation by upregulating the expression of the oncogenic factor c‐Myc.^6^ Additionally, SLC transporters also remodel the tumour microenvironment (TME) by regulating metabolite transportation to and from the microenvironment.^3^ Physiologically, SLC transporters maintain metabolic homeostasis by forming complex transporter networks.^7^ However, most studies have focused only on the existing SLC transporters of several drug targets because of the complex structure and function of SLC transporters and limitations of existing technologies.^8^, ^9^, ^10^ Therefore, the expression profiles of the SLC transporter family members should be comprehensively analysed from a holistic perspective to explore their association with tumour microenvironment heterogeneity and clinical prognosis.

According to global data, gastric cancer is currently the third leading cause of cancer‐related fatalities.^11^ Owing to the biological heterogeneity of gastric cancer, clinical outcomes vary significantly, even among patients with identical Tumour Node Metastasis (TNM) stages and comparable treatment regimens.^12^, ^13^ Some potential predictors contributing to the benefits of chemotherapy and prognosis prediction have been identified in previous studies^14^; however, these biomarkers have not been applied clinically, and further research and validation are required. New biomarkers must be developed and validated to promote accurate diagnosis and treatment. In this study, we comprehensively explored the expression profiles of SLC family genes and identified two distinct SLC subtypes, SLC‐S1 and SLC‐S2. A model comprising 12 genes was established to quantify SLC subtypes in gastric cancer, which was named as the SLC score. Furthermore, we evaluated the association between both SLC subtypes and the SLC score model with microenvironmental characteristics, clinical outcomes and treatment responses in different cohorts of gastric cancers.

## 2 MATERIALS AND METHODS

### 2.1 Bulk transcriptomic data collection and processing

Transcriptome data and clinical information for patients with gastric cancer were obtained from the Gene Expression Omnibus (GEO) database, The Cancer Genome Atlas (TCGA), and the UCSC Xena website. The GSE62254 and GSE26942 datasets were retrieved from the GEO database, and TCGA‐STAD data were obtained from the TCGA database (Tables S1 and S2). The GSE62254 dataset was preprocessed and corrected using the ‘affy’ and ‘simpleaffy’ R packages, whereas standardized data from the GSE26942 dataset were directly used. For the TCGA‐STAD transcriptome data, fragments per kilobase of transcript per million (FPKM) data were downloaded and converted into transcripts per million (TPM) data. Since the survival outcomes of stage IV gastric cancer are influenced by various factors, including tumor heterogeneity, distant metastasis, therapeutic interventions, and severe complications, data for stage IV patients in the three cohorts were removed, and the corresponding SLC family gene expression matrices were extracted. Ultimately, these data were merged using the ‘combat’ function in the ‘sva’ R package. Additionally, data from the GSE57303 cohort were downloaded separately for further analysis.

### 2.2 Identification of SLC subtypes

Gastric cancer samples were classified using the ‘CancerSubtype’ R package. Non‐negative matrix factorization, a powerful method for class discovery and dimension reduction,^15^ was used to identify SLC subtypes by distinguishing molecular patterns from SLC gene transcriptome data.

### 2.3 TME deconvolution and biological process analysis

The single‐sample gene set enrichment analysis algorithm included in the ‘GSVA’ R package was used to deconvolve the TME cells. Based on published literature, we assembled 23 gene sets representing myeloid, lymphocyte and stromal cell types to determine the infiltration level of each cell type. The infiltration level was determined by measuring the enrichment fraction of the gene set in the sample output from gene expression profiling‐based single‐sample gene set enrichment analysis. Additionally, we conducted a biological process analysis using GSVA against 50 hallmark biological pathways obtained from the Molecular Signature Database (http://www.gsea‐msigdb.org).

### 2.4 Multiomics analysis

We performed a comprehensive multiomics investigation consisting of analyses of copy number variation (CNV), somatic genetic mutations, DNA methylation and proteomics.^16^ We acquired masked copy number segment data using the ‘TCGAbiolinks’ R package and used GISTIC 2.0, with Genepattern as the default setting, to analyse the CNV fragments. Using the ‘TCGAbiolinks’ R package, we downloaded the gene mutation file and identified significantly mutated genes (SMGs) using the MutSigCV algorithm (*q* < 0.05).^17^ We conducted a chi‐square test to analyse differences in the mutation frequency of the top 100 SMGs between SLC‐S1 and SLC‐S2. For DNA methylation analysis, we downloaded level 3 DNA methylation (Methylation450k) data from the UCSC Xena database and preprocessed them using the ‘ChAMP’ R package. We assigned DNA methylation values to each gene, mapped the median *β* value of the probe to the promoter region, and selected 1000 genes with the largest methylation value (*β* value) variability as candidate genes. The Kruskal–Wallis test was performed to identify differentially methylated genes between SLC‐S1 and SLC‐S2. Additionally, we explored changes in the transcriptional expression of the abovementioned differentially methylated genes between SLC‐S1 and SLC‐S2, examined the correlation between transcriptional expression and methylation levels of each gene, and calculated *p*‐values adjusted for multiple tests using the Benjamini–Hochberg correction method. Finally, significantly varied methylation genes (SVMGs) were defined according to the following criteria: significant variation in the methylation value and corresponding transcription expression value among different groups (adjusted *p*‐value <0.05) and negative correlation of the methylation value with the transcription expression value of the SVMG (adjusted *p*‐value <0.05).

### 2.5 Generation of the SLC score

The SLC score was calculated as described. First, we employed the ‘limma’ R package to analyse differentially expressed genes (DEGs) between SLC‐S1 and SLC‐S2 in the GSE62254 cohort. The criteria were set such that DEGs with an absolute value of log_2_ fold‐change >1 and an adjusted *p*‐value of <0.01 would be selected. We reduced the dimensionality of the identified DEGs via the Boruta algorithm in the ‘Boruta’ R package for clusters A and B of the up‐ and down‐regulated gene populations of SLC‐S2, respectively. We set the parameters as follows: doTrace = 2, maxRuns = 100 and nTree = 500.^18^ The prognostic function of the genes was identified using LASSO‐Cox regression analysis of the dimensionality‐reduced genes. The selected genes were used to develop an SLC scoring model, which was calculated as follows: SLC score = (average expression of selected gene cluster A)–(average expression of selected gene cluster B).

### 2.6 Therapeutic response prediction

We used the CTRP database (https://portals.broadinstitute.org/ctrp) to predict drug sensitivity. CTRP provides sensitivity data for 860 human cancer cell lines (CCLs) to 481 compounds. The area under the dose–response curve (AUC) was used to calculate drug sensitivity, with a lower AUC indicating higher sensitivity. Additionally, tumour immune dysfunction and exclusion (TIDE; http://tide.dfci.harvard.edu) was used to predict the immune checkpoint inhibitor (ICI) response for each tumour sample.^19^ To identify potential therapeutic drugs, we analysed patient molecular data by determining DEGs between SLC‐S2 and SLC‐S1, or between high and low SLC scores, followed by CMap analysis. We used the principle of CMap analysis to identify drugs with lower scores and higher theoretical efficacy in managing specific diseases.^20^, ^21^

### 2.7 Statistical analysis

Student's *t*‐test and Mann–Whitney U test were used to compare parametric and nonparametrically distributed continuous variables between the two groups, respectively. Chi‐square and Fisher's exact tests were used to analyse categorical variables. For normally distributed continuous variables, Pearson's correlation tests were used to assess correlations among the variables, whereas Spearman's correlation tests were used for non‐normally distributed continuous variables. We employed Kaplan–Meier analysis to generate survival curves and used the log‐rank test to determine statistical significance. In addition, we used univariate and multivariate Cox regression models to calculate hazard ratios for univariate and multivariate analyses, respectively. All statistical analyses were performed using SPSS (version 25.0; SPSS, Inc) and R software (version 4.0.2; The R Project for Statistical Computing). Statistical significance was considered at a two‐sided *p*‐value of less than 0.05.

## 3 RESULTS

### 3.1 Identification of molecular subtypes mediated by SLC family genes

**FIGURE 1:**
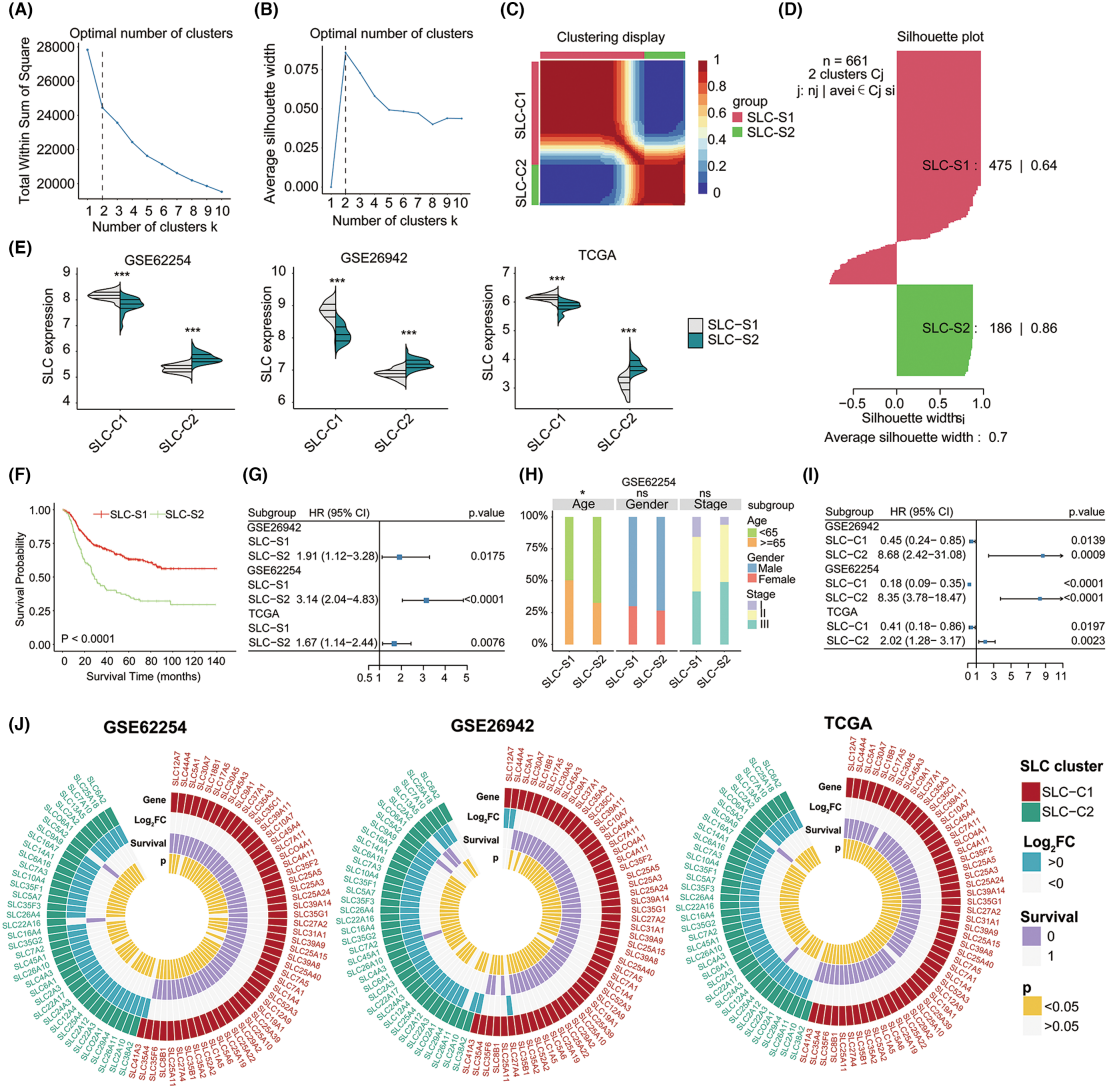
Identification of SLC subtypes in gastric cancer and their impact on clinical prognosis. The optimal number of clusters was determined using the Elbow method (A) and Silhouette method (B) from the FactoExtra R package. (C) The heatmap showed that gastric cancer samples (661 patients) were clustered by using the non‐negative matrix factorization method. (D) Silhouette width plots were generated using the CancerSubtypes package to evaluate the quality of the clustering. (E) Violin plots exhibited the distribution of the expression levels of the SLC gene cluster in patients with different SLC subtypes in the GSE62254 (left, 222 patients), GSE26942 (middle, 150 patients) and TCGA‐STAD (right, 289 patients) cohorts, respectively. Student's *t*‐test was used to determine significance. *p < 0.05, **p < 0.01, ***p < 0.001. (F) Survival analysis was used to evaluate different survival patterns between SLC subtypes. (G) Forest plot was used to display the impact of different SLC subtypes on the overall survival (OS) of patients with gastric cancer in the GSE62254 (222 patients), GSE26942 (150 patients) and TCGA‐STAD (289 patients) cohorts, respectively. Each small square represents an unadjusted risk ratio, while the horizontal line represents the corresponding 95% confidence interval (CI). (H) The stacked plot was displayed to show the clinical features of different SLC subtypes in the GSE62254 cohort (222 patients). Statistical significance was assessed using the chi‐square test. *p < 0.05, **p < 0.01, ***p < 0.001. (I) Forest plot showed the impact of different SLC gene clusters on the overall survival (OS) of patients with gastric cancer in the GSE62254 (222 patients), GSE26942 (150 patients) and TCGA‐STAD (289 patients) cohorts, respectively. Each small square represents an unadjusted risk ratio, while the horizontal line represents the corresponding 95% confidence interval (CI). (J) Circus plots were displayed to show the differences in the expression of genes in the different SLC gene clusters of different SLC subtypes and their impact on prognosis. Log_2_FC is a value obtained by comparing the expression level of a gene in the SLC‐S1 subtype and SLC‐S2 subtype. When Log_2_FC >0, it means that the gene is upregulated in the SLC‐S2 subtype, whereas when Log_2_FC <0, it means that the gene is downregulated in the SLC‐S2 subtype. Survival = 0 means that the expression level of the gene is a protective factor for the patient's prognosis, while survival = 1 means that the expression level of the gene is a risk factor for the patient's prognosis. A *p*‐value of less than 0.05 indicates that there is a significant difference in the effect of the gene's expression level on the prognosis. Student's *t*‐test was used to determine significance. SLC‐S1, SLC subtype 1; SLC‐S2, SLC subtype 2; SLC‐C1, SLC gene cluster 1; SLC‐C2, SLC gene cluster2; CI, confidence interval; HR, hazard ratios; Log_2_FC, Log_2_Fold Change.

Figure S1 illustrated the workflow used in this study. The SLC family gene members included in the typing analysis were screened prior to the study. Initially, the expression matrices of the SLC family genes (count >1) from the GSE62254, GSE26942 and TCGA‐STAD cohorts, comprising 354 members, were merged. Survival‐related SLC genes were screened using the Cox regression model from the ‘CancerSubtypes’ R package, resulting in the selection of 89 SLC genes for subsequent analysis (Table S3). We used the Elbow and Silhouette methods from the FactoExtra R package (Figure 1A, B), and the K‐means and ward D methods from the NbClust R package (Figure S2A,B), to confirm that *k* = 2 is the optimal number of clusters. Subsequently, non‐negative matrix factorization (NMF) was employed to conduct clustering analysis, resulting in the classification of three cohorts into two distinct subtypes (Figure 1C): SLC subtype 1 (SLC‐S1) and SLC subtype 2 (SLC‐S2). The silhouette width value of 0.7 (Figure 1D) showed that the gastric cancer samples matched well with their designated subtypes. Simultaneously, the expression patterns of the SLC family gene members were classified into two groups: SLC gene cluster 1 (SLC‐C1) and SLC gene cluster 2 (SLC‐C2) (Figure 1C and Table S3). The heatmap (Figure 1C) and violin plots (Figure 1E) showed that the expression level of SLC‐C1 was remarkably higher in patients with SLC‐S1 than in those with SLC‐S2, whereas the expression level of SLC‐C2 in patients with SLC‐S1 was considerably lower than that in patients with SLC‐S2.

Survival analysis revealed that SLC‐S1 significantly improved overall survival (OS) (Figure 1F). Similarly, patients with stage SLC‐S2 had a significantly increased risk of recurrence (Figure S2C) and death (Figure 1G) in the three independent cohorts when compared with those with SLC‐S1. The stacked plots (Figure 1H, Figure S2D,E) illustrated the distribution of clinical features in the two SLC subtypes. In the three cohorts, the distributions of sex and TNM stage, except for age, did not differ significantly between the two subtypes. Furthermore, multivariate Cox regression analysis was conducted to assess the influence of clinical characteristics and SLC subtypes on clinical outcomes. SLC subtypes in the GSE62254 cohort highly correlated with OS (Figure S2F) and relapse‐free survival (RFS) (Figure S2G), even after adjusting for clinical characteristics.

The correlation heatmap (Figure S2H) revealed a positive correlation between the genes in SLC‐C1; similar findings were observed for the genes in SLC‐C2. However, the SLC‐C1 and SLC‐C2 genes were negatively correlated. Notably, genes within SLC‐C2 were indicative of poor prognosis, and their expression was significantly elevated in SLC‐S2. By contrast, genes within the SLC‐C1 predicted a relatively good prognosis (RFS and OS) and were significantly upregulated in SLC‐S1 (Figures S1I,J, S2I).

### 3.2 Biological pathway activation and TME cell infiltration traits of different SLC subtypes

**FIGURE 2:**
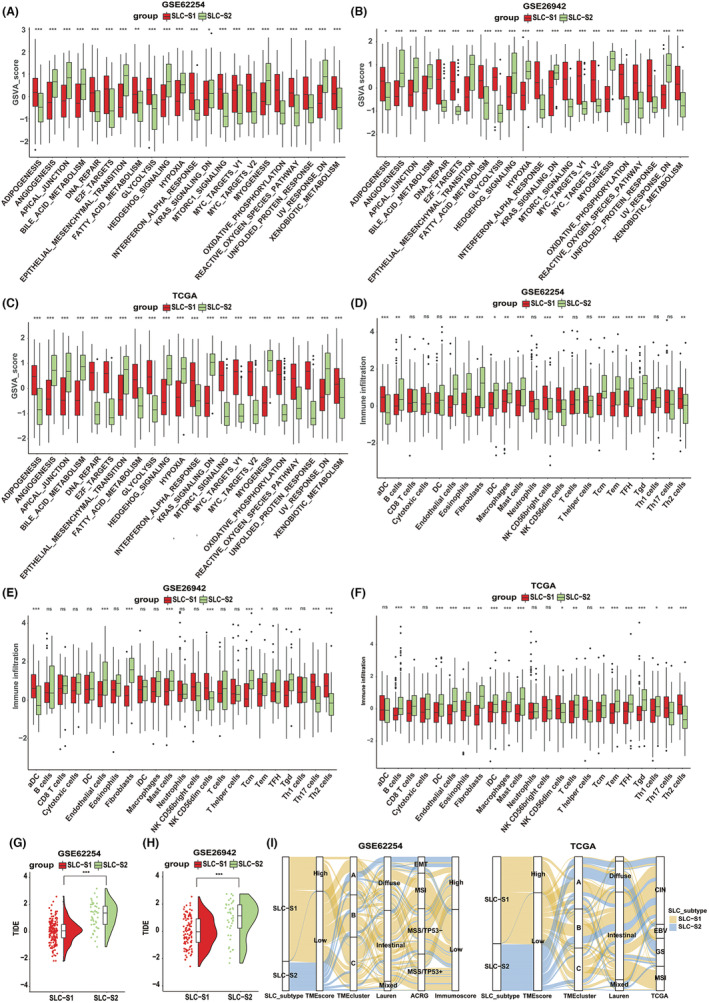
The relationship between SLC subtypes and tumour microenvironment and biological function characteristics. Box plots depicted the activation status of biological pathways of different SLC subtypes based on hallmark gene set in the (A) GSE62254 (222 patients), (B) GSE26942 (150 patients) and (C) TCGA‐STAD (289 patients) cohorts. Another set of box plots exhibited the tumour microenvironment landscape of different SLC subtypes in the (D) GSE62254 (222 patients), (E) GSE26942 (150 patients) and (F) TCGA‐STAD (289 patients) cohorts. The TIDE algorithm was used to predict the TIDE values for SLC‐S1 and SLC‐S2 in the (G) GSE62254 (222 patients) and (H) GSE26942 (150 patients) cohorts and the results were shown in violin plots. (I) Sankey diagram of different SLC subtypes corresponding to different molecular subtypes in the GSE62254 (left, 222 patients) and TCGA‐STAD (right, 182 patients) cohorts. The box represents 25%–75% of the value, the line in the box represents the median, the whisker represents 1.5 interquartile spacing, and the black dot represents the outlier. Student's *t*‐test was utilized to evaluate statistical significance. *p < 0.05, **p < 0.01, ***p < 0.001. EMT, epithelial‐mesenchymal transition; SLC‐S1, SLC subtype 1; SLC‐S2, SLC subtype 2.

Box plots (Figure 2A–C) and heatmaps (Figure S3A) indicated that cell cycle‐related pathways, including DNA repair, E2F signalling and the MYC signalling pathways, were highly activated in SLC‐S1, but significantly suppressed in SLC‐S2, across all three cohorts. Elevation in the GSVA scores of the angiogenesis, epithelial–mesenchymal transition (EMT), Hh signalling and myogenesis pathways implied increased activation of the stromal and immune‐inflammatory pathways in SLC‐S2. Furthermore, some metabolic pathways were significantly enriched in SLC‐S1.

In terms of TME cell infiltration characteristics, the infiltration abundances of Th2 cells, endothelial cells and fibroblasts were significantly higher in SLC‐S2 than in SLC‐S1 (Figure 2D–F, Figure S3B). Consequently, we predicted that the TME feature of SLC‐S2 reflects ‘immune exclusion’, a hallmark characterized by stromal cell infiltration that inhibits infiltration of cytotoxic immune cells into the tumour parenchyma from the stroma surrounding tumour cell nests. Previous studies demonstrated that patients with ‘immune exclusion’ often exhibit a poor response to immune checkpoint inhibitors (ICIs).^22^ The TIDE algorithm forecasts the response rates of patients to ICIs, and a higher TIDE score indicates an immune escape phenotype and unfavourable response to ICIs in patients with cancer.^19^ As expected, patients with SLC‐S2 exhibited higher TIDE scores than those with SLC‐S1 in the GSE62254 and GSE26942 cohorts (Figure 2G, H). Finally, we investigated the correlation between SLC subtypes and various other molecular subtypes. The graphs in Figure 2I revealed that patients with SLC‐S2 from the GSE62254 and TCGA‐STAD cohorts were mostly concentrated in the specific molecular subtypes characterized by stromal hyperactivation.

### 3.3 Multiomics analysis of samples in different SLC subtypes

**FIGURE 3:**
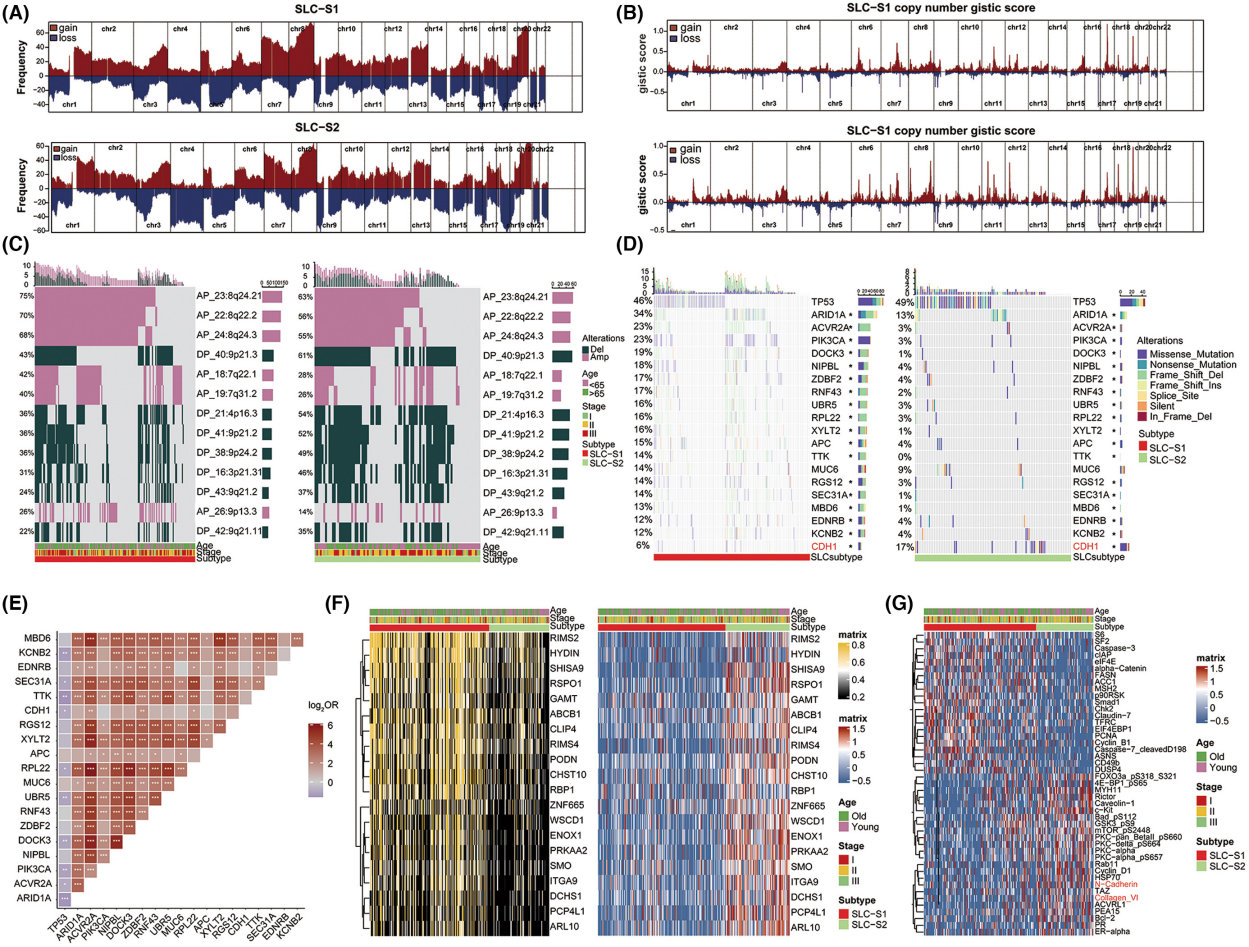
The copy number variation, genomic alteration and methylation features within the SLC subtypes. Copy number variation (A) frequency and (B) Gistic score were used to identify the gains and losses in different SLC genotypes. The colours red and blue represented copy number gain and copy number loss, respectively. (C) Oncoprints depicted the copy number variation events that differed significantly across different SLC subtypes. (D) Oncoprints depicted the significantly mutated genes (SMGs) corresponding to different SLC subtypes within the TCGA‐STAD cohort of 289 patients. Cohort details and SLC subtypes are used as sample annotations of the heatmaps. (E) Mutual exclusivity of the top 20 significant mutated genes was demonstrated by the heatmap, where Log_2_OR <0 indicated mutual exclusivity (‘competition’ occurred), and Log_2_OR >0 represented co‐occurrence analysis (‘coexistence’). (F) Heatmaps showed the significantly varied methylation genes (SVMG) among different SLC subtypes at methylation level (left) and transcriptome level (right) in the TCGA‐STAD cohort (278 patients). (G) Heatmap exhibited the landscape of differentially expressed proteins in different SLC subtypes in the TCGA‐STAD cohort (230 patients). SLC‐S1, SLC subtype 1; SLC‐S2, SLC subtype 2; SMG, significantly mutated gene; SVMG, significantly varied methylation gene.

We performed a multiomics analysis of the TCGA‐STAD cohort to understand the biological attributes of various SLC subtypes. We observed a striking resemblance between SLC‐S1 and SLC‐S2 in terms of the distribution of CNV events (Figure 3A,B), followed by the identification of 13 copy number alteration events with significant difference using chi‐square test (Figure 3C). These events occur mainly on chromosomes 9, 8, 7, 4 and 3. We observed a higher frequency of copy number deletion events in SLC‐S2, but a lower frequency of copy number amplification events in SLC‐S2 than in SLC‐S1. Analysis of somatic mutations revealed a distinct difference in the mutation rates of the 78 SMGs between SLC‐S1 and SLC‐S2 (Table S4 and Figure 3D). Compared to SLC‐S2, the mutation rate of these SMGs mostly increased in SLC‐S1, except for *CDH1* (E‐cadherin). As shown in Figure 3E, most SMGs with increased mutation rates in SLC‐S1 were commensal. Additionally, we explored the differences in the degree of gene promoter methylation and corresponding transcriptional expression levels between SLC‐S1 and SLC‐S2. We identified 313 SVMGs (Table S5); the top 20 are shown in Figure 3F. Most SVMGs were hypermethylated in SLC‐S1 and had low transcriptional expression levels. By contrast, these SVMGs were hypomethylated in SLC‐S2 and had high transcriptional expression levels. Ultimately, protein data from the TCGA‐STAD cohort revealed significant overexpression of EMT‐associated proteins (N‐cadherin and collagen‐VI) in SLC‐S2 (Figure 3G).

### 3.4 Construction and evaluation of SLC score

**FIGURE 4:**
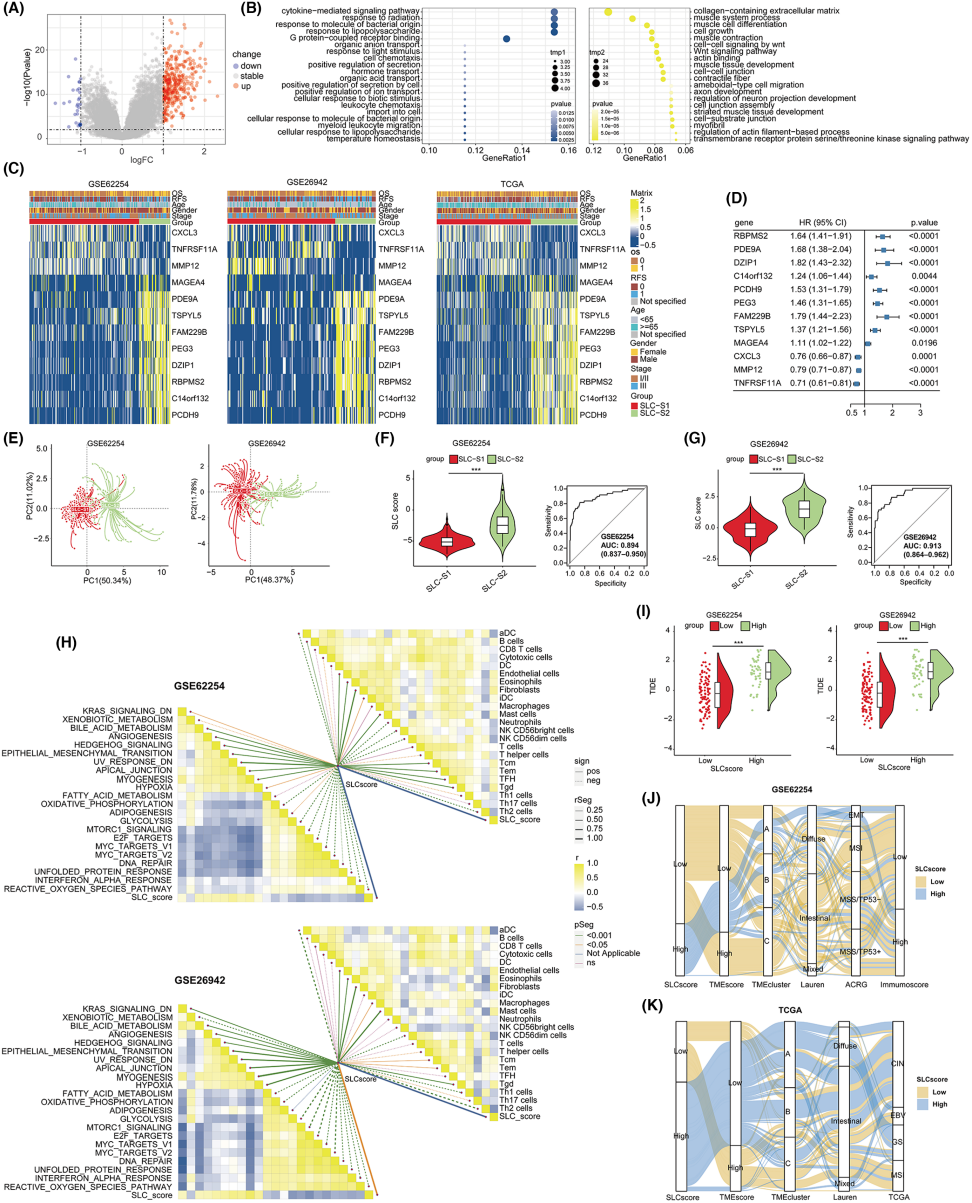
Construction and exploration of the SLC score in gastric cancer. (A) The volcano plot displayed the differentially expressed genes (DEGs) between patients with SLC‐S1 and SLC‐S2 subtypes in the GSE62254 cohort. (B) Gene Ontology analysis illustrated the biological pathways enriched by downregulated genes (left) and upregulated genes (right), respectively. (C) Heatmap exhibited the expression of genes consisting of SLC score in different SLC subtypes in the GSE62254 (left, 222 patients), GSE26942 (middle, 150 patients), and TCGA‐STAD (right, 289 patients) cohorts, respectively. Cohort details and SCS subtypes are used as sample annotations of the heatmaps. (D) The forest plot showed the associations between the expression of genes consisting of SLC score and OS of patients in the GSE62254 cohort (222 patients). Each small square represents an unadjusted risk ratio, while the horizontal line represents the corresponding 95% confidence interval (CI). (E) The principal component analyses demonstrated the effective distinction of patients with SLC‐S1 and SLC‐S2 subtypes based on the SLC score in GSE62254 (left, 222 patients) and GSE26942 (right, 150 patients) cohorts. Violin plots (F and G left) of SLC score value in two SLC subtypes, and receiver operating characteristics curve (F and G right) of SLC score for the prediction of SLC‐S2 subtype in the GSE62254 (222 patients) and GSE26942 (150 patients) cohorts. Boxes inside the violins represent 25%–75% of values, lines in boxes represent median values, whiskers represent 1.5 interquartile ranges and black dots represent outliers. Student's *t*‐test was utilized to evaluate statistical significance. *p < 0.05, **p < 0.01, ***p < 0.001. (H) The correlation matrix showed the correlation between the SLC score and infiltration of TME cells, as well as the correlation between the SLC score and activation levels of biological pathways in the GSE62254 (top, 222 patients) and GSE26942 (bottom, 150 patients) cohorts. ‘pos’ represented positive relation; ‘neg’ represented negative relation; ‘rSeg’ represented the absolute value of correlation coefficient; ‘r’ represented correlation coefficient. (I) The TIDE algorithm was used to predict the TIDE values for subgroups with low or high SLC scores in the GSE62254 (left, 222 patients) and GSE26942 (right, 150 patients) cohorts, and the results were shown in violin plots. Student's *t*‐test was utilized to evaluate statistical significance. *p < 0.05, **p < 0.01, ***p < 0.001. (J and K) Sankey diagram of subgroups with low or high SLC scores corresponding to different molecular subtypes in the GSE62254 (222 patients) and TCGA‐STAD (182 patients) cohorts. SLC‐S1, SLC subtype 1; SLC‐S2, SLC subtype 2; CI, confidence interval; HR, hazard ratios.

Considering the unfavourable prognosis and limited response to immunotherapy observed in patients with SLC‐S2, our primary objective was to develop a concise scoring tool that enables precise identification of this specific patient subgroup. We selected the GSE62254 dataset as the training set owing to its larger patient population when compared to GSE26942. Furthermore, GSE62254 offered a broader range of patient information when compared to the other two datasets. Our screening process revealed 381 DEGs in SLC‐S2 according to the screening criteria (Table S6, Figure 4A). Gene ontology (GO) analysis of the abovementioned DEGs revealed that upregulated genes in SLC‐S2 were predominantly involved in biological processes related to matrix activation (Figure 4B). Next, using the Boruta algorithm and LASSO‐Cox regression analysis, we selected 12 genes associated with SLC subtypes as gene signatures of SLC‐S2 and used them to construct a scoring model to quantify SLC gene typing, named as the ‘SLC score’ (Figure 4C). The SLC score was determined using the following formula: SLC score = (*RBPMS2 + PDE9A + DZIP1 + C14orf132 + PCDH9 + PEG3 + FAM229B + TSPYL5 + MAGEA4*)/9‐(*CXCL3 + MMP12 + TNFRSF11A*)/3. Among these 12 genes, nine were highly expressed in SLC‐S2 and acted as risk factors for patient recurrence and poor prognosis, whereas the other three were expressed at low levels in SLC‐S2, and their expression levels significantly negatively correlated with the risk of recurrence and poor prognosis (Figure S4A,D). Principal component analysis showed that patients with SLC‐S1 and SLC‐S2 were accurately distinguished based on their SLC scores (Figure 4E, Figure S4B). Furthermore, the median SLC scores of patients with SLC‐S2 were markedly higher than those of patients with SLC‐S1 (Figure 4F, G, Figure S4C (left)). Receiver operating characteristic curve analysis confirmed that the SLC score was reliable for distinguishing SLC‐S2, with diagnostic accuracy rates of 0.894, 0.913 and 0.900 in the GSE62254, GSE26942 and TCGA‐STAD cohorts, respectively (Figure 4F, G, Figure S4C (right)).

The correlation tests (Figure 4H, Figure S4D) revealed a significant positive correlation between the SLC score, activation level of stromal‐related pathways, and degree of stromal cell infiltration. Using the ‘survminer’ R package, we split the patients into high‐ and low‐SLC‐score groups (minprop was set to 0.3). Further analysis revealed that patients with high SLC scores had remarkably higher TIDE scores in all three cohorts (Figure 4I, Figure S4E), indicating a lower response rate to ICIs. Figure 4J,K show enrichment of low‐ and high‐SLC‐score groups in other molecular subtypes, which aligns with previously observed enrichment of SLC‐S2 in other molecular types. These findings may have implications for patient selection and treatment strategies.

### 3.5 Prognostic role of the SLC scores in patients with gastric cancer

**FIGURE 5:**
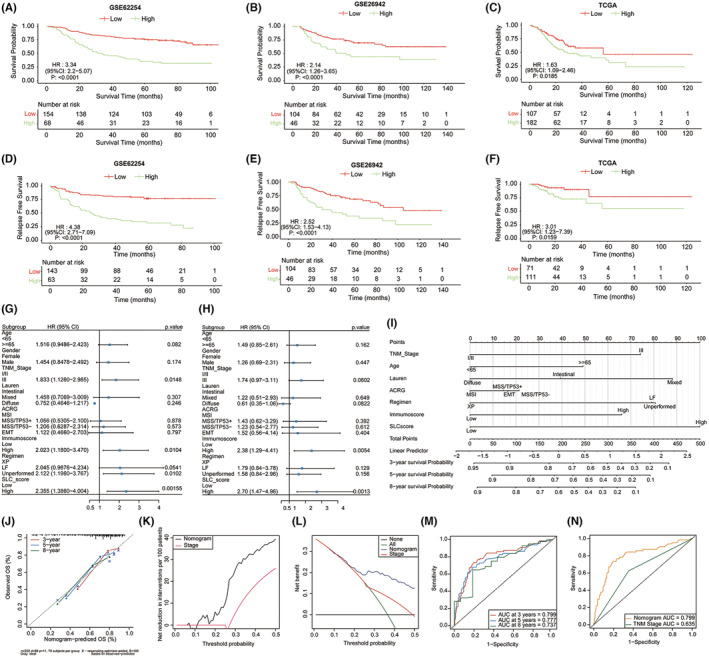
Relationship between SLC score and clinical prognosis. Kaplan–Meier curves of OS in (A) GSE62254 (222 patients), (B) GSE26942 (150 patients), and (C) TCGA‐STAD cohorts (289 patients) according to the SLC score. Kaplan–Meier curves of RFS in (D) GSE62254 cohort (206 patients), (E) GSE26942 (150 patients) and (F) TCGA‐STAD (201 patients) cohorts according to the SLC score. The log‐rank test was utilized to compare the statistical significance. (G) Multivariate survival analyses for OS (G) and (H) RFS of SLC score, clinical variables and some molecule types in the GSE62254 cohort (RFS: 206 patients, OS: 222 patients). Each small square represents an unadjusted risk ratio, while the horizontal line represents the corresponding 95% confidence interval (CI). (I) A nomogram predicting the survival probability for patients with gastric cancer in the GSE62254 cohort. (J) Calibration plot of the nomogram model. (K) and (L) Decision curve analysis for the nomogram model. (M) Receiver operating characteristics curve of nomogram model for the prediction of survival at 3, 5 and 8 years, respectively. (N) Receiver operating characteristics curve of nomogram model and TNM stage for the prediction of survival at 3 years. SLC‐S1, SLC subtype 1; SLC‐S2, SLC subtype 2; CI, confidence interval; HR, hazard ratios.

We investigated the prognostic value of the SLC scores in patients with gastric cancer. In the training cohort, patients with high SLC scores had significantly lower OS rates than those with low SLC scores (Figure 5A). Similar results were obtained for GSE26942 (Figure 5B), GSE57303 (Figure S5) and TCGA‐STAD (Figure 5C). Additionally, patients with high SLC scores also showed a remarkably increased risk of recurrence in the GSE62254, GSE26942 and TCGA‐STAD cohorts (Figure 5D–F). After adjusting for clinical characteristics using multivariate Cox regression analysis, a high SLC score was independently associated with a worse OS (Figure 5G) and RFS (Figure 5H). Combining the SLC score with TNM staging in a nomogram improved the accuracy of prognosis prediction when compared to that of TNM staging alone (Figure 5I‐L). Furthermore, we assessed the prognostic accuracy of the nomogram using time‐dependent receiver operating characteristic analysis in the GSE62254 cohort, yielding areas under the curve of 0.799, 0.777 and 0.737 for predicting the 3‐, 5‐ and 8‐year OS, respectively (Figure 5M). Comparison of areas under the curve of the nomogram and TNM staging for predicting 3‐year OS revealed superior performance using the nomogram (0.799 vs. 0.635; Figure 5N).

### 3.6 Correlation between SLC subtype, SLC score and adjuvant chemotherapy response

**FIGURE 6:**
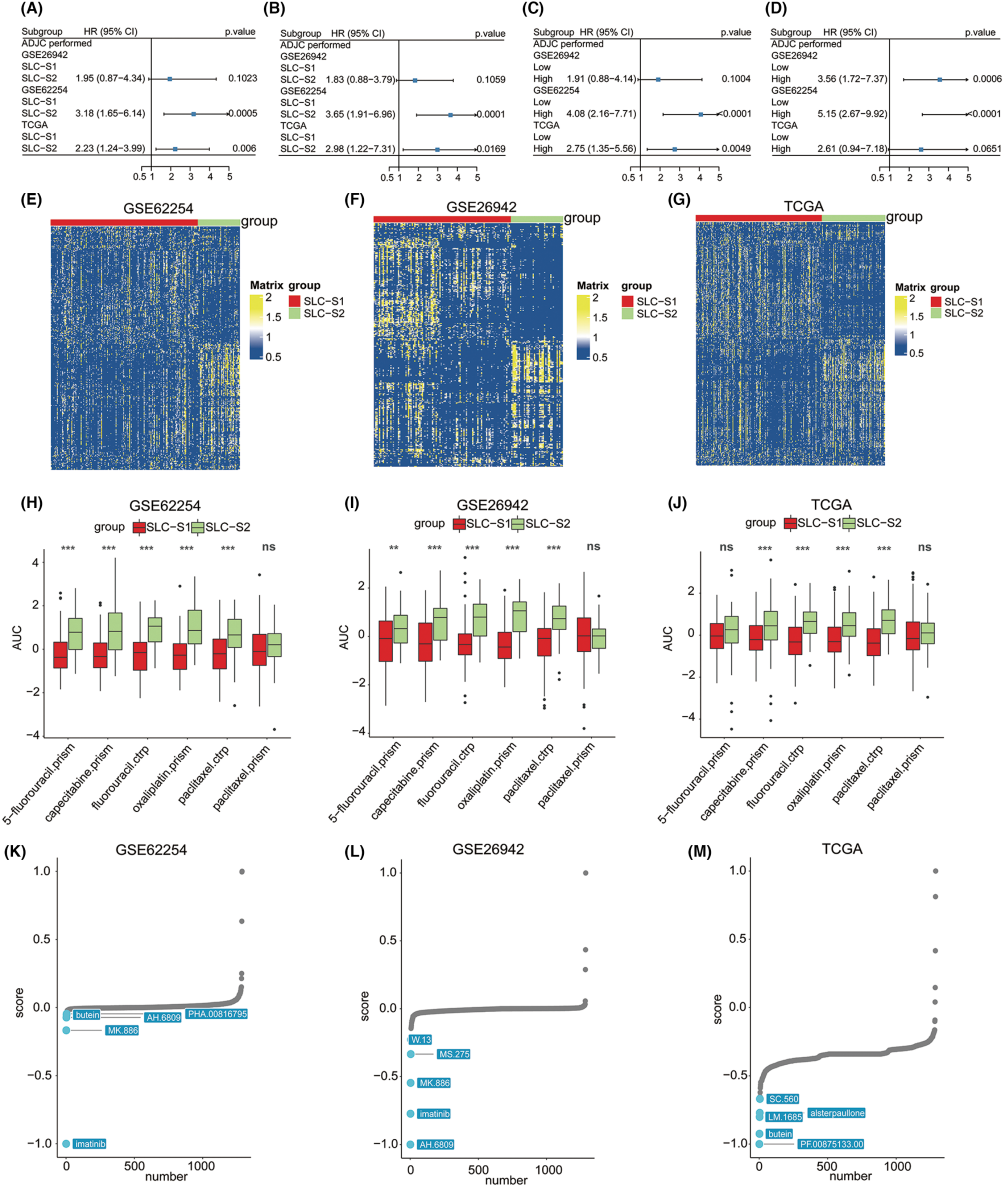
Relationship between the SLC subtype, SLC score and chemotherapy response. (A) Forest plot was used to display the impact of different SLC subtypes on overall survival (OS) of patients with gastric cancer receiving ADJC in the GSE62254 (222 patients), GSE26942 (150 patients) and TCGA‐STAD (289 patients) cohorts, respectively. (B) Forest plot was used to display the impact of different SLC subtypes on relapse‐free survival (RFS) of patients with gastric cancer receiving ADJC in the GSE62254 (206 patients), GSE26942 (150 patients) and TCGA‐STAD (201 patients) cohorts, respectively. (C) Forest plot was used to display the impact of low or high SLC score on overall survival (OS) of patients with gastric cancer receiving ADJC in the GSE62254 (222 patients), GSE26942 (150 patients) and TCGA‐STAD (289 patients) cohorts, respectively. (D) Forest plot was used to display the impact of low or high SLC score on relapse‐free survival (RFS) of patients with gastric cancer receiving ADJC in the GSE62254 (206 patients), GSE26942 (150 patients) and TCGA‐STAD (201 patients) cohorts, respectively. Based on CTRP analysis, heatmaps exhibited predicted AUC values of different compounds corresponding to patients with different SLC subtypes in the (E) GSE62254 (222 patients), (F) GSE26942 (150 patients) and (G) TCGA‐STAD (289 patients) cohorts, the lower the AUC value is, the higher the sensitivity is. Based on CTRP analysis, violin plots exhibited predicted AUC values of common chemotherapy drugs corresponding to patients with different SLC subtypes in the (H) GSE62254 (222 patients), (I) GSE26942 (150 patients) and (J) TCGA‐STAD (289 patients) cohorts. Student's *t*‐test was utilized to evaluate statistical significance. *p < 0.05, **p < 0.01, ***p < 0.001. The top five drugs with the lowest CMAP scores corresponding to patients with SLC‐S2 were demonstrated in the (K) GSE62254 (222 patients), (L) GSE26942 (150 patients) and (M) TCGA‐STAD (289 patients) cohorts, respectively. SLC‐S1, SLC subtype 1; SLC‐S2, SLC subtype 2; CI, confidence interval; HR, hazard ratios.

Considering that adjuvant chemotherapy may affect patient prognosis, we analysed the survival of patients with different SLC subtypes after adjuvant chemotherapy (ADJC). The forest plot indicated that ADJC increased the risk of death (Figure 6A) and recurrence (Figure 6B) in patients with SLC‐S2 compared with that in patients with SLC‐S1. We also examined the prognostic value of the SLC score to evaluate the benefits of chemotherapy in patients with gastric cancer. The findings indicated that ADJC increased the risk of death (Figure 6C) and recurrence (Figure 6D) in patients with high SLC scores than in patients with low SLC scores. To further explore the patients' response to chemotherapy, we examined the sensitivity of patients with gastric cancer to commonly used chemotherapy drugs in the three cohorts using the CTRP database. Heatmaps were generated to show the distribution of the area under the curve of compounds based on the CTRP database in patients with different SLC subtypes (Figure 6E–G) and patients with different SLC scores (Figure S6A–C). The findings revealed that patients with SLC‐S2 or high SLC scores were less sensitive to chemotherapeutic drugs than those with SLC‐S1 (Figure 6H–J) or low SLC scores (Figure S6D–F).

Based on CMAP analysis, we explored drugs with the potential to treat patients with gastric cancer who have SLC‐S2 or a high SLC score. Imatinib, AH.6809 and butein were promising drug candidates for treating patients with SLC‐S2 (Figure 6K–M). Further investigation revealed that imatinib was the most promising drug candidate for patients with high SLC scores (Figure S6G–I).

## 4 DISCUSSION

SLC transporters are involved in many basic physiological functions, including nutrient uptake, ion influx or efflux and waste disposal, all of which are closely related to many human diseases.^7^, ^23^ Increasing evidence has shown that SLC transporter dysregulation is involved in the occurrence and progression of gastric cancer.^5^, ^24^, ^25^ Our study revealed that survival‐related SLC family gene members comprise two distinct coexpression clusters (SLC‐C1 and SLC‐C2), and high expression levels of SLC‐C1 predicted better prognosis, whereas high expression levels of SLC‐C2 predicted poor prognosis. Based on the transcriptome of survival‐related SLC family genes, we identified novel molecular subtypes (SLC‐S1 and SLC‐S2) of gastric cancer that remarkedly correlated with different clinical outcomes, TME landscape and biological pathways. Among them, the SLC‐S2 subtype was characterized by high concentrations of SLC‐C2 cluster, enrichment of stromal cells and activation of stromal pathways, which was linked to a poor response to chemotherapy and immunotherapy in previous studies.^3^, ^26^ By contrast, patients with SLC‐S2 had the worst RFS and OS, and insensitive responses to chemotherapy and immunotherapy.

Notably, the frequency of copy number deletion events was significantly higher in SLC‐S2 than that in SLC‐S1. These events in SLC‐S2 were primarily occurred in chromosomes 9p21, 9q21, 4p16, 9p24 and 3p21. Chromosomal deletions in cancer have been suggested to be related to a poor prognosis.^27^ Previous studies showed that chromosome 9p21 is among the most frequently deleted regions in several human cancers, particularly in melanoma and small‐cell lung cancer.^27^, ^28^ Chromosome 3p deletions are present in almost all small‐cell lung cancers and approximately 90% of non‐small cell lung cancers.^29^ Therefore, deletion of tumour suppressors in the affected chromosomal regions may contribute to the poor clinical outcomes observed in SLC‐S2. Notably, we found that the mutation rate of most high frequency mutated genes in SLC‐S1 was significantly higher than that in SLC‐S2, which may explain the relatively poor response of SLC‐S2 to immunotherapy. E‐cadherin, a tumour suppressor participating in regulating cell proliferation, differentiation, apoptosis and migration, promotes EMT and tumour metastasis in gastric cancer when its expression is lost.^30^ These findings are consistent with our finding that the mutation rate of E‐cadherin was significantly increased in SLC‐S2. Furthermore, most hypermethylated genes in SLC‐S1 exhibited a pattern of hypomethylation and high transcriptional expression in SLC‐S2, indicating that hypomethylation of these genes plays an important role in the biological features of SLC‐S2.

Patients with SLC‐S2 exhibit a poor prognosis and response to chemotherapy and immunotherapy; therefore, it is critical to develop a tool that can identify patients with SLC‐S2. To this end, we identified a set of 12 candidate genes using Boruta and LASSO‐Cox regression analysis and established a predictive model named as the ‘SLC score’. We also validated the SLC score using multiple independent cohorts and found that it can accurately identify patients with SLC‐S2. Furthermore, the SLC score is an independent prognostic factor for gastric cancer and can predict the response of patients with gastric cancer to chemotherapy and immunotherapy. Additionally, some genes used to model SLC scores have also been studied in gastric and other cancers. Particularly, high expression levels of *RBPMS2*,^31^ *DZIP1*,^32^ *PEG3*,^33^ *TSPYL5* ^34^ and *MAGEA4* ^35^, ^36^ in gastric cancer or other tumour tissues have been associated with a poor prognosis, while high expression levels of *CXCL3* and *TNFRSF11A* in gastric cancer have been associated with a better prognosis^37^, ^38^; these observations are consistent with our findings. Thus, the clinical translational potential of SLC family gene members can be realized using the SLC score, which has good reproducibility and clinical applicability for predicting prognosis and guiding therapeutic strategies. In conclusion, the specific biological functions and potential mechanisms of these genes in the SLC scoring model of gastric cancer require further investigation.

Previous studies have demonstrated that SLCs regulate resistance mechanisms to chemotherapy and targeted therapy drugs.^3^, ^39^ A specific study reported that among 435 effect‐mediating targets, 12 SLCs can serve as drug targets, increasing the interest of researchers in investigating SLCs as drug targets for antitumor therapy.^1^, ^40^ Various in vitro and in vivo experiments have validated the potential of SLC inhibitors, such as JPH203, AZD3965, BAY‐8002 and 7ACC2, to alter tumour cell metabolism and impede tumour growth.^9^, ^41^, ^42^ Unfortunately, clinical trials of these drugs have not yielded substantial results. Furthermore, the structural features of SLC membrane proteins are relatively complex, fully expressed and redundant in terms of substrate specificity, which pose a major challenge to the development of tumour therapeutic drugs targeting SLC transporters.^8^, ^43^ We screened the CMAP database to identify molecules that can treat SLC‐S2 gastric cancer and identified imatinib as one of the most promising drugs. Imatinib, a specific tyrosine kinase inhibitor that targets the BCR‐ABL, KIT and PDGFR receptors, is used to treat various cancers, including but not limited to chronic myeloid leukaemia and gastrointestinal stromal tumours.^44^ Previous studies reported that imatinib can reverse gastric cancer resistance to taxanes by selectively depleting CLIP‐170S.^45^ Therefore, imatinib showed potential as a drug for treating patients with SLC‐S2 and may contribute to chemosensitization. Further in vivo and in vitro preclinical studies are required to validate the tumour‐suppressive effects of imatinib in gastric cancer.

This study had some limitations. First, not all 456 SLC genes were included in our analysis. Therefore, it inevitably carries the risk that some SLC transporters with critical pathophysiological functions and clinical significance may have been filtered out. Second, the potential of the SLC score for predicting gastric cancer prognosis and treatment response was evaluated using a retrospective database with inherent selection bias, thereby necessitating further validation through prospective studies. Finally, the cut‐off values for the SLC score must be standardized in future prospective studies.

In conclusion, we systematically revealed the heterogeneity of clinical behaviour, TME and biological behaviour associated with the transcriptome signature and clinical characteristics of SLC family genes. Additionally, the SLC score, designed to identify SLC‐S2, is a translatable model that can effectively predict poor prognosis and insensitive responses to chemotherapy and immunotherapy in patients with gastric cancer. The SLC score can supplement TNM staging evaluation and provide more accurate guidance for the comprehensive treatment of gastric cancer.

## AUTHOR CONTRIBUTIONS

**Yue Zhang:** Data curation (equal); formal analysis (equal); investigation (equal); methodology (equal); software (equal); visualization (equal); writing – original draft (equal). **Zhihong Liu:** Data curation (equal); formal analysis (equal); investigation (equal); methodology (equal); software (equal); visualization (equal); writing – original draft (equal). **Lingbo Li:** Data curation (equal); formal analysis (equal); investigation (equal); methodology (equal); software (equal); visualization (equal); writing – original draft (equal). **Dongqiang Zeng:** Writing – review and editing (supporting). **Huiying Sun:** Writing – review and editing (supporting). **Jianhua Wu:** Writing – review and editing (supporting). **Rui Zhou:** Conceptualization (equal); funding acquisition (supporting); project administration (equal); resources (equal); supervision (equal). **Wangjun Liao:** Conceptualization (lead); funding acquisition (lead); project administration (lead); resources (lead); supervision (lead).

## FUNDING INFORMATION

This work was supported by the National Natural Science Foundation of China (No. 82102731 to RZ, No. 82073303 to WL), Natural Science Foundation of Guangdong Province of China (2020A1515110686 to RZ, 2022A1515012418 to RZ), and President Foundation of Nanfang Hospital, Southern Medical University (2020C020 to RZ).

## CONFLICT OF INTEREST STATEMENT

The authors declare no conflict of interest.

## Supporting information


Figure S1

Figure S2

Figure S3

Figure S4

Figure S5

Figure S6

Table S1

Table S2

Table S3

Table S4

Table S5

Table S6

Captions Figure and Table S1‐S6

## Data Availability

This study analysed publicly available datasets, which can be found at: GSE62254 (http://www.ncbi.nlm.nih.gov/geo/query/acc.cgi?acc=GSE62254); GSE26942 (http://www.ncbi.nlm.nih.gov/geo/query/acc.cgi?acc=GSE26942); GSE57303 (http://www.ncbi.nlm.nih.gov/geo/query/acc.cgi?acc=GSE57303); TCGA‐STAD (https://xenabrowser.net/datapages/?cohort=TCGA).

## References

[1] Panda S , Banerjee N , Chatterjee S . Solute carrier proteins and c‐Myc: a strong connection in cancer progression. Drug Discov Today. 2020;25(5):891‐900. 32105718 10.1016/j.drudis.2020.02.007

[2] Hediger MA , Clemencon B , Burrier RE , et al. The ABCs of membrane transporters in health and disease (SLC series): introduction. Mol Aspects Med. 2013;34(2–3):95‐107. 23506860 10.1016/j.mam.2012.12.009 PMC3853582

[3] Rashid K , Ahmad A , Liang L , Liu M , Cui Y , Liu T . Solute carriers as potential oncodrivers or suppressors: their key functions in malignant tumor formation. Drug Discov Today. 2021;26(7):1689‐1701. 33737072 10.1016/j.drudis.2021.03.004

[4] El‐Gebali S , Bentz S , Hediger MA , et al. Solute carriers (SLCs) in cancer. Mol Aspects Med. 2013;34(2–3):719‐734. 23506905 10.1016/j.mam.2012.12.007

[5] Liu X , Li T , Ma Z , et al. SLC26A9 deficiency causes gastric intraepithelial neoplasia in mice and aggressive gastric cancer in humans. Cell Oncol (Dordr). 2022;45(3):381‐398. 35426084 10.1007/s13402-022-00672-x PMC9187568

[6] Ye W , Chen C , Gao Y , et al. Overexpression of SLC34A2 is an independent prognostic indicator in bladder cancer and its depletion suppresses tumor growth via decreasing c‐Myc expression and transcriptional activity. Cell Death Dis. 2017;8(2):e2581. 28151475 10.1038/cddis.2017.13 PMC5386463

[7] Song W , Li D , Tao L , Luo Q , Chen L . Solute carrier transporters: the metabolic gatekeepers of immune cells. Acta Pharm Sin B. 2020;10(1):61‐78. 31993307 10.1016/j.apsb.2019.12.006 PMC6977534

[8] Cesar‐Razquin A , Snijder B , Frappier‐Brinton T , et al. A call for systematic research on solute carriers. Cell. 2015;162(3):478‐487. 26232220 10.1016/j.cell.2015.07.022

[9] Wang N , Jiang X , Zhang S , et al. Structural basis of human monocarboxylate transporter 1 inhibition by anti‐cancer drug candidates. Cell. 2021;184(2):370‐383. e13. 33333023 10.1016/j.cell.2020.11.043

[10] Halestrap AP . The SLC16 gene family—structure, role and regulation in health and disease. Mol Aspects Med. 2013;34(2–3):337‐349. 23506875 10.1016/j.mam.2012.05.003

[11] Thrift AP , El‐Serag HB . Burden of gastric cancer. Clin Gastroenterol Hepatol. 2020;18(3):534‐542. 31362118 10.1016/j.cgh.2019.07.045 PMC8859863

[12] Zhou R , Wu Z , Zhang J , et al. Clinical significance of accurate identification of lymph node status in distant metastatic gastric cancer. Oncotarget. 2016;7(1):1029‐1041. 26556854 10.18632/oncotarget.6009 PMC4808049

[13] Jiang Y , Zhang Q , Hu Y , et al. ImmunoScore signature: a prognostic and predictive tool in gastric cancer. Ann Surg. 2018;267(3):504‐513. 28002059 10.1097/SLA.0000000000002116

[14] Chia NY , Tan P . Molecular classification of gastric cancer. Ann Oncol. 2016;27(5):763‐769. 26861606 10.1093/annonc/mdw040

[15] Xu T , Le TD , Liu L , et al. CancerSubtypes: an R/Bioconductor package for molecular cancer subtype identification, validation and visualization. Bioinformatics. 2017;33(19):3131‐3133. 28605519 10.1093/bioinformatics/btx378

[16] Zhou R , Li L , Xi S , et al. Expression pattern of secretory‐cell‐related transcriptional signatures in colon adenocarcinomas defines tumor microenvironment characteristics and correlates with clinical outcomes. Mol Oncol. 2023;17(3):499‐517. 36349418 10.1002/1878-0261.13338 PMC9980301

[17] Lawrence MS , Stojanov P , Polak P , et al. Mutational heterogeneity in cancer and the search for new cancer‐associated genes. Nature. 2013;499(7457):214‐218. 23770567 10.1038/nature12213 PMC3919509

[18] Zhou R , Xie F , Liu K , et al. Cross talk between acetylation and methylation regulators reveals histone modifier expression patterns posing prognostic and therapeutic implications on patients with colon cancer. Clin Epigenetics. 2022;14(1):70. 35606881 10.1186/s13148-022-01290-y PMC9128235

[19] Jiang P , Gu S , Pan D , et al. Signatures of T cell dysfunction and exclusion predict cancer immunotherapy response. Nat Med. 2018;24(10):1550‐1558. 30127393 10.1038/s41591-018-0136-1 PMC6487502

[20] Yang C , Zhang H , Chen M , et al. A survey of optimal strategy for signature‐based drug repositioning and an application to liver cancer. Elife. 2022;11:e71880. 35191375 10.7554/eLife.71880 PMC8893721

[21] Musa A , Ghoraie LS , Zhang SD , et al. A review of connectivity map and computational approaches in pharmacogenomics. Brief Bioinform. 2018;19(3):506‐523. 28069634 10.1093/bib/bbw112 PMC5952941

[22] Hegde PS , Chen DS . Top 10 challenges in cancer immunotherapy. Immunity. 2020;52(1):17‐35. 31940268 10.1016/j.immuni.2019.12.011

[23] Schumann T , Konig J , Henke C , et al. Solute carrier transporters as potential targets for the treatment of metabolic disease. Pharmacol Rev. 2020;72(1):343‐379. 31882442 10.1124/pr.118.015735

[24] Zhang JX , Xu Y , Gao Y , et al. Retraction note: decreased expression of miR‐939 contributes to chemoresistance and metastasis of gastric cancer via dysregulation of SLC34A2 and Raf/MEK/ERK pathway. Mol Cancer. 2022;21(1):227. 36577974 10.1186/s12943-022-01702-w PMC9795626

[25] Shimakata T , Kamoshida S , Kawamura J , et al. Immunohistochemical expression profiles of solute carrier transporters in alpha‐fetoprotein‐producing gastric cancer. Histopathology. 2016;69(5):812‐821. 27245475 10.1111/his.13004

[26] Hugo W , Zaretsky JM , Sun L , et al. Genomic and transcriptomic features of response to anti‐PD‐1 therapy in metastatic melanoma. Cell. 2016;165(1):35‐44. 26997480 10.1016/j.cell.2016.02.065 PMC4808437

[27] Chen M , Yang Y , Liu Y , et al. The role of chromosome deletions in human cancers. Adv Exp Med Biol. 2018;1044:135‐148. 29956295 10.1007/978-981-13-0593-1_9

[28] Sulong S , Moorman AV , Irving JA , et al. A comprehensive analysis of the CDKN2A gene in childhood acute lymphoblastic leukemia reveals genomic deletion, copy number neutral loss of heterozygosity, and association with specific cytogenetic subgroups. Blood. 2009;113(1):100‐107. 18838613 10.1182/blood-2008-07-166801

[29] Zabarovsky ER , Lerman MI , Minna JD . Tumor suppressor genes on chromosome 3p involved in the pathogenesis of lung and other cancers. Oncogene. 2002;21(45):6915‐6935. 12362274 10.1038/sj.onc.1205835

[30] Bure IV , Nemtsova MV , Zaletaev DV . Roles of E‐cadherin and noncoding RNAs in the epithelial‐mesenchymal transition and progression in gastric cancer. Int J Mol Sci. 2019;20(12):2870. 31212809 10.3390/ijms20122870 PMC6627057

[31] Zhao H , Tong Y , Pan S , Qiu Z , Liu P , Guo P . RBPMS2, as a novel biomarker for predicting lymph node metastasis, guides therapeutic regimens in gastric cancer [J]. Hum Cell. 2022;35(2):599‐612. 34981466 10.1007/s13577-021-00667-0

[32] Gong Y , Liu X , Sahu A , Reddy AV , Wang H . Exploration of hub genes, lipid metabolism, and the immune microenvironment in stomach carcinoma and cholangiocarcinoma. Ann Transl Med. 2022;10(15):834. 36034995 10.21037/atm-22-3530 PMC9403925

[33] Luo YD , Liu XY , Fang L , et al. Mutant Kras and mTOR crosstalk drives hepatocellular carcinoma development via PEG3/STAT3/BEX2 signaling. Theranostics. 2022;12(18):7903‐7919. 36451866 10.7150/thno.76873 PMC9706580

[34] Sun Q , Guo D , Li S , et al. Combining gene expression signature with clinical features for survival stratification of gastric cancer. Genomics. 2021;113(4):2683‐2694. 34129933 10.1016/j.ygeno.2021.06.018

[35] Davari K , Holland T , Prassmayer L , et al. Development of a CD8 co‐receptor independent T‐cell receptor specific for tumor‐associated antigen MAGE‐A4 for next generation T‐cell‐based immunotherapy. J Immunother Cancer. 2021;9(3):e002035. 33771892 10.1136/jitc-2020-002035 PMC7996660

[36] Brisam M , Rauthe S , Hartmann S , et al. Expression of MAGE‐A1‐A12 subgroups in the invasive tumor front and tumor center in oral squamous cell carcinoma. Oncol Rep. 2016;35(4):1979‐1986. 26820613 10.3892/or.2016.4600

[37] Li Y , He X , Fan L , Zhang X , Xu Y , Xu X . Identification of a novel immune prognostic model in gastric cancer. Clin Transl Oncol. 2021;23(4):846‐855. 32857339 10.1007/s12094-020-02478-5

[38] Xiao Z , Nie K , Han T , et al. Development and validation of a TNF family‐based signature for predicting prognosis, tumor immune characteristics, and immunotherapy response in colorectal cancer patients. J Immunol Res. 2021;2021:6439975. 34541005 10.1155/2021/6439975 PMC8448595

[39] Gottesman MM , Fojo T , Bates SE . Multidrug resistance in cancer: role of ATP‐dependent transporters. Nat Rev Cancer. 2002;2(1):48‐58. 11902585 10.1038/nrc706

[40] Rask‐Andersen M , Almen MS , Schioth HB . Trends in the exploitation of novel drug targets. Nat Rev Drug Discov. 2011;10(8):579‐590. 21804595 10.1038/nrd3478

[41] Kanai Y . Amino acid transporter LAT1 (SLC7A5) as a molecular target for cancer diagnosis and therapeutics. Pharmacol Ther. 2022;230:107964. 34390745 10.1016/j.pharmthera.2021.107964

[42] Polanski R , Hodgkinson CL , Fusi A , et al. Activity of the monocarboxylate transporter 1 inhibitor AZD3965 in small cell lung cancer. Clin Cancer Res. 2014;20(4):926‐937. 24277449 10.1158/1078-0432.CCR-13-2270 PMC3929348

[43] Wang W , Zou W . Amino acids and their transporters in T cell immunity and cancer therapy. Mol Cell. 2020;80(3):384‐395. 32997964 10.1016/j.molcel.2020.09.006 PMC7655528

[44] Kim JL , Lee DH , Jeong S , et al. Imatinib‐induced apoptosis of gastric cancer cells is mediated by endoplasmic reticulum stress. Oncol Rep. 2019;41(3):1616‐1626. 30569109 10.3892/or.2018.6945 PMC6365688

[45] Thakkar PV , Kita K , Castillo UD , et al. CLIP‐170S is a microtubule +TIP variant that confers resistance to taxanes by impairing drug‐target engagement. Dev Cell. 2021;56(23):3264‐3275. e7. 34672971 10.1016/j.devcel.2021.09.023 PMC8665049

