# Supplementary figures and images for: Co‐expression pattern of SLC transporter genes associated with the immune landscape and clinical outcomes in gastric cancer
Source: J Cell Mol Med. 2023 Nov 1;27(24):4181–94. doi: 10.1111/jcmm.18003 (PMC10746955; doi:10.1111/jcmm.18003)

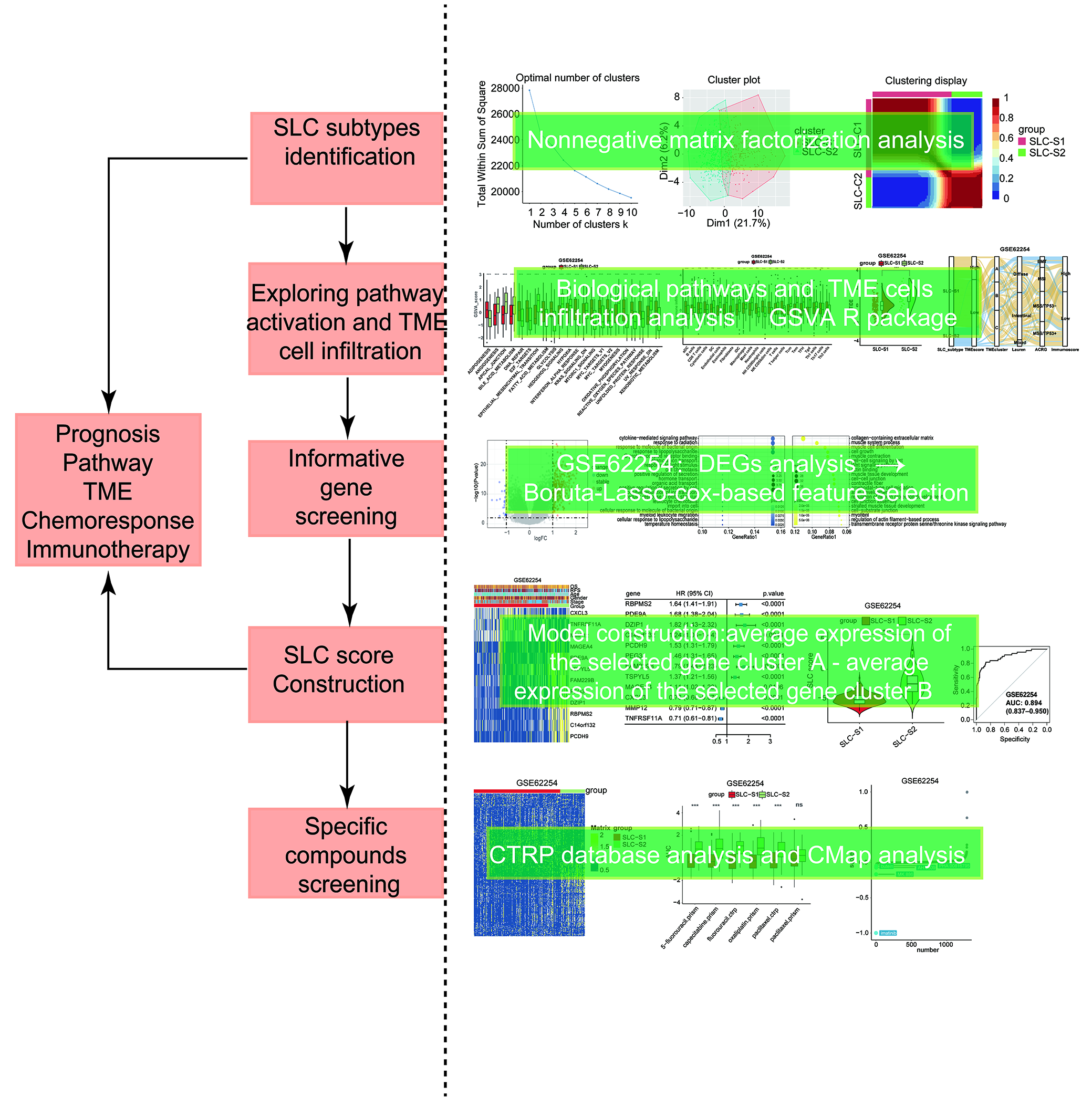

Supplement: Supplementary file 1 — Figure S1 [file JCMM-27-4181-s003.tif]

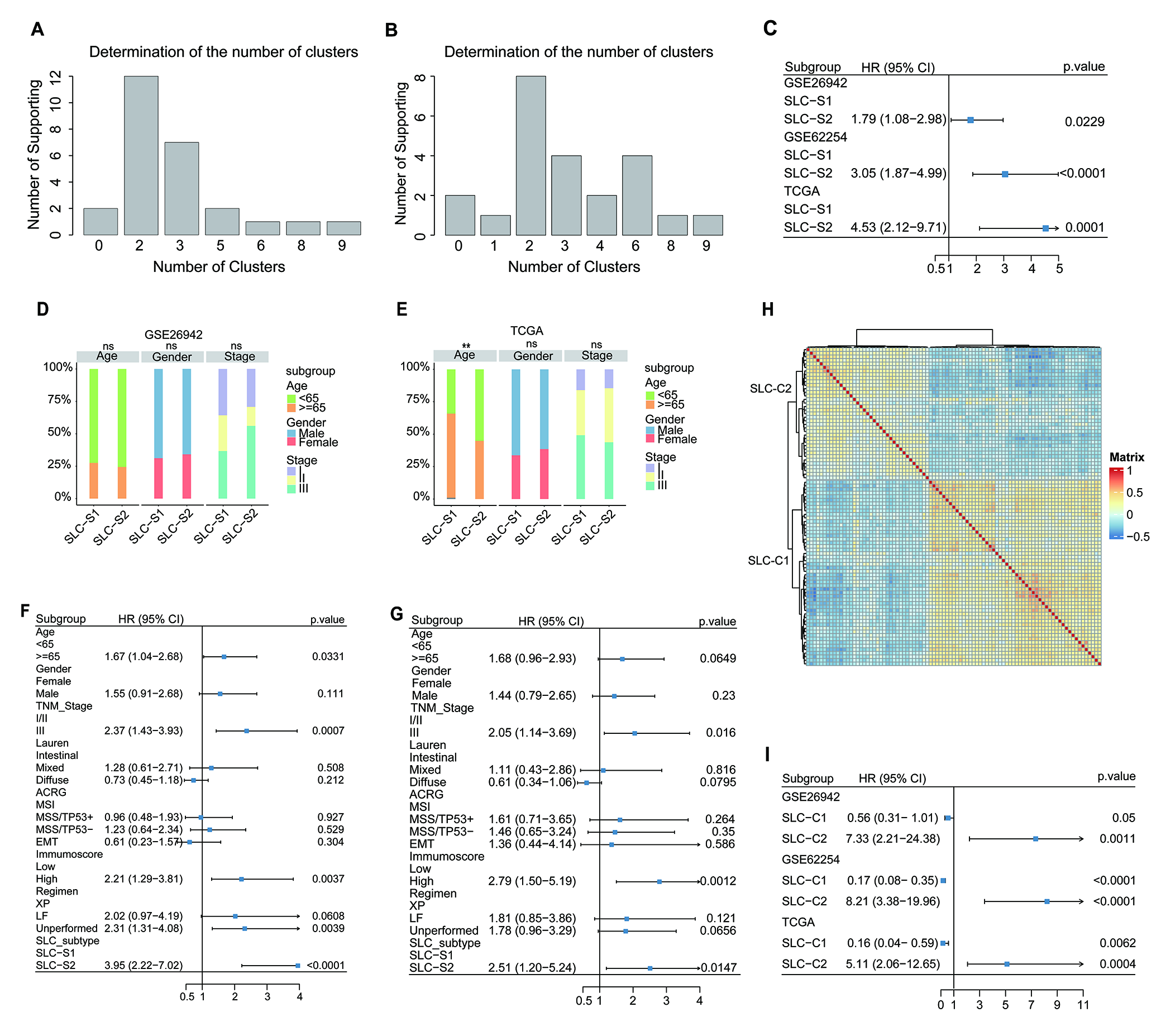

Supplement: Supplementary file 2 — Figure S2 [file JCMM-27-4181-s012.tif]

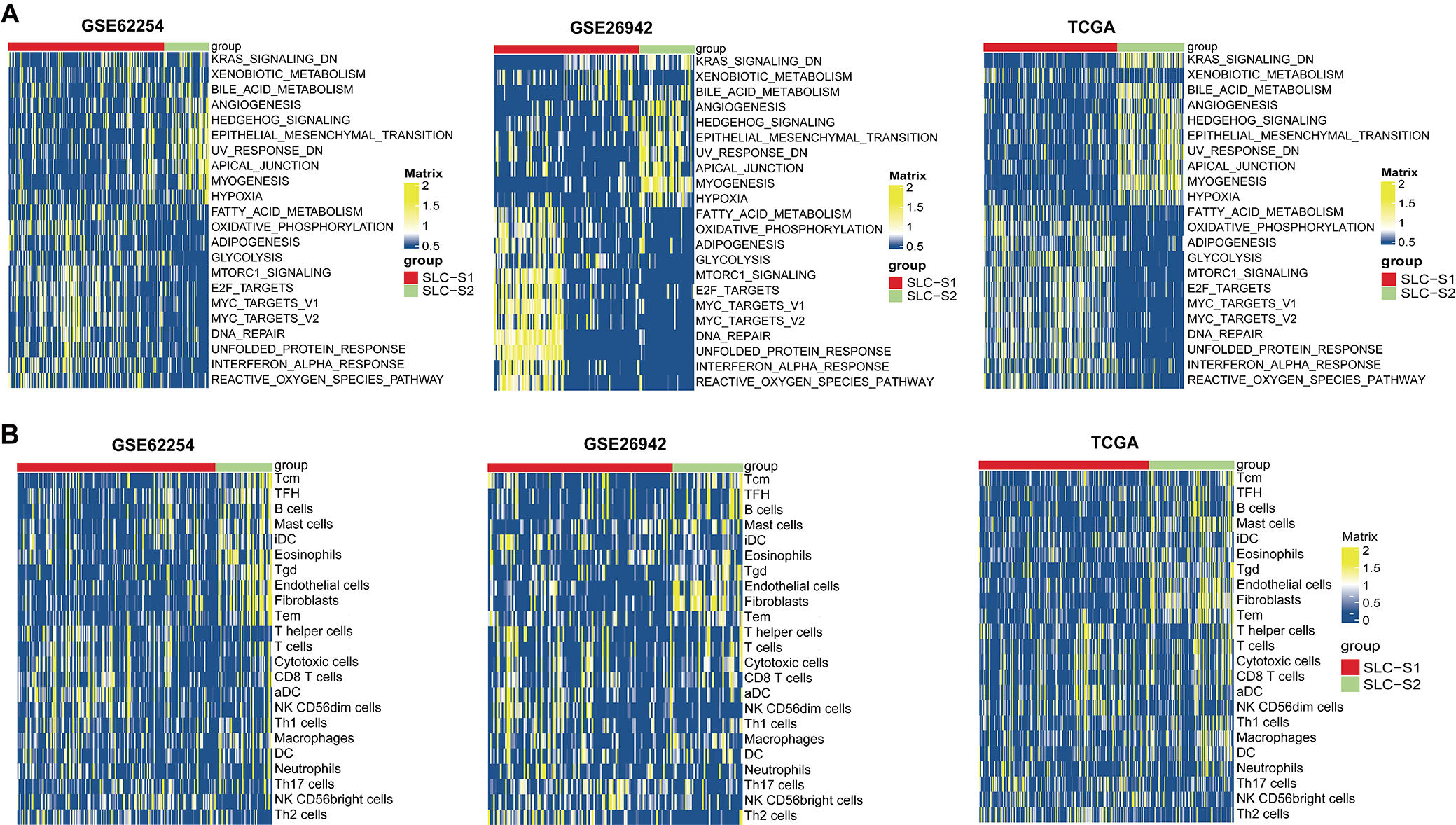

Supplement: Supplementary file 3 — Figure S3 [file JCMM-27-4181-s009.tif]

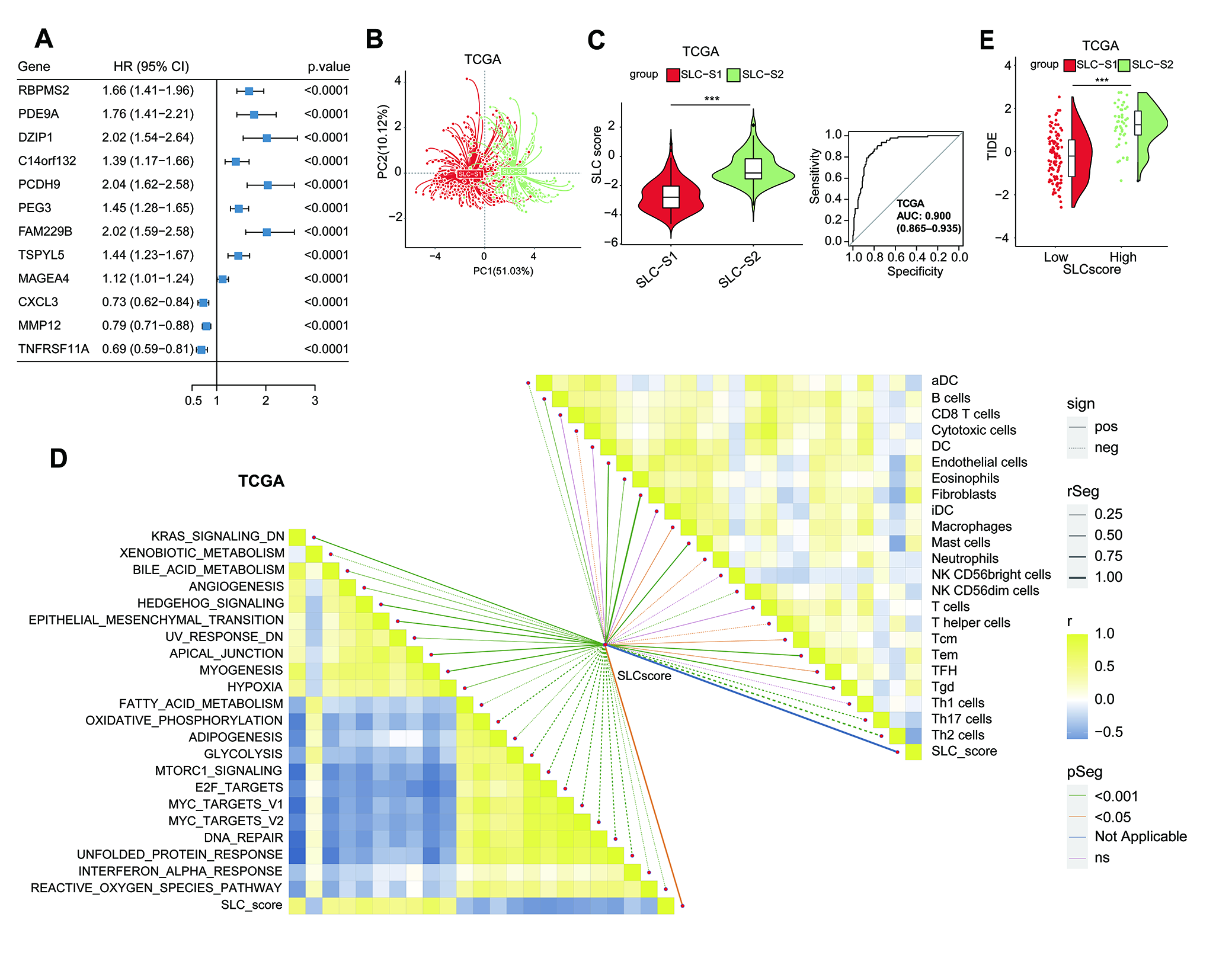

Supplement: Supplementary file 4 — Figure S4 [file JCMM-27-4181-s006.tif]

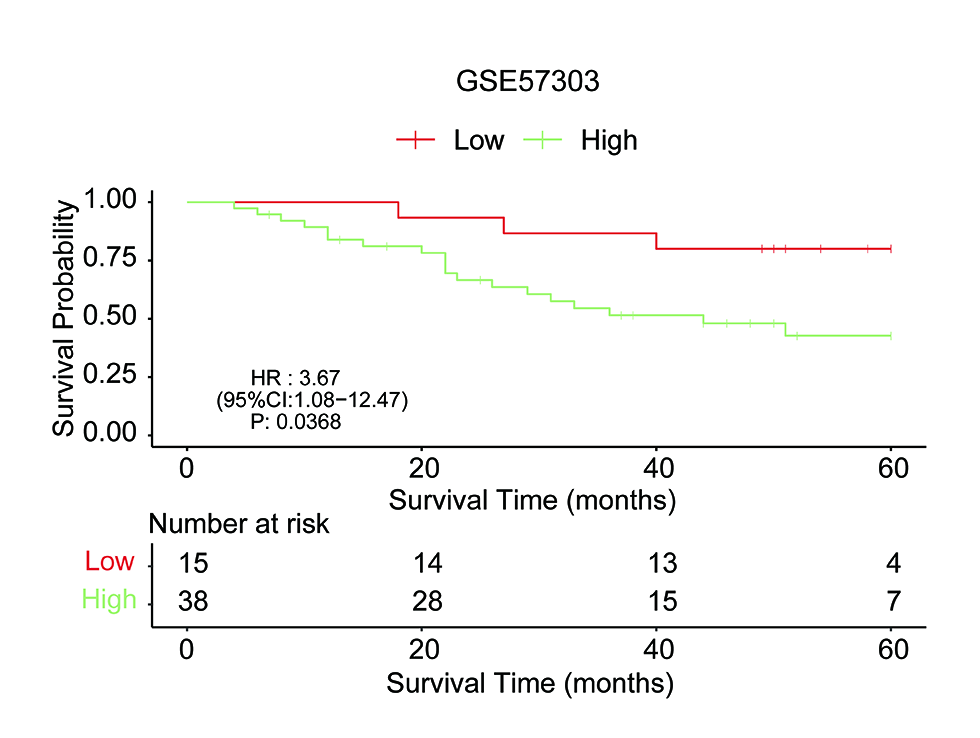

Supplement: Supplementary file 5 — Figure S5 [file JCMM-27-4181-s010.tif]

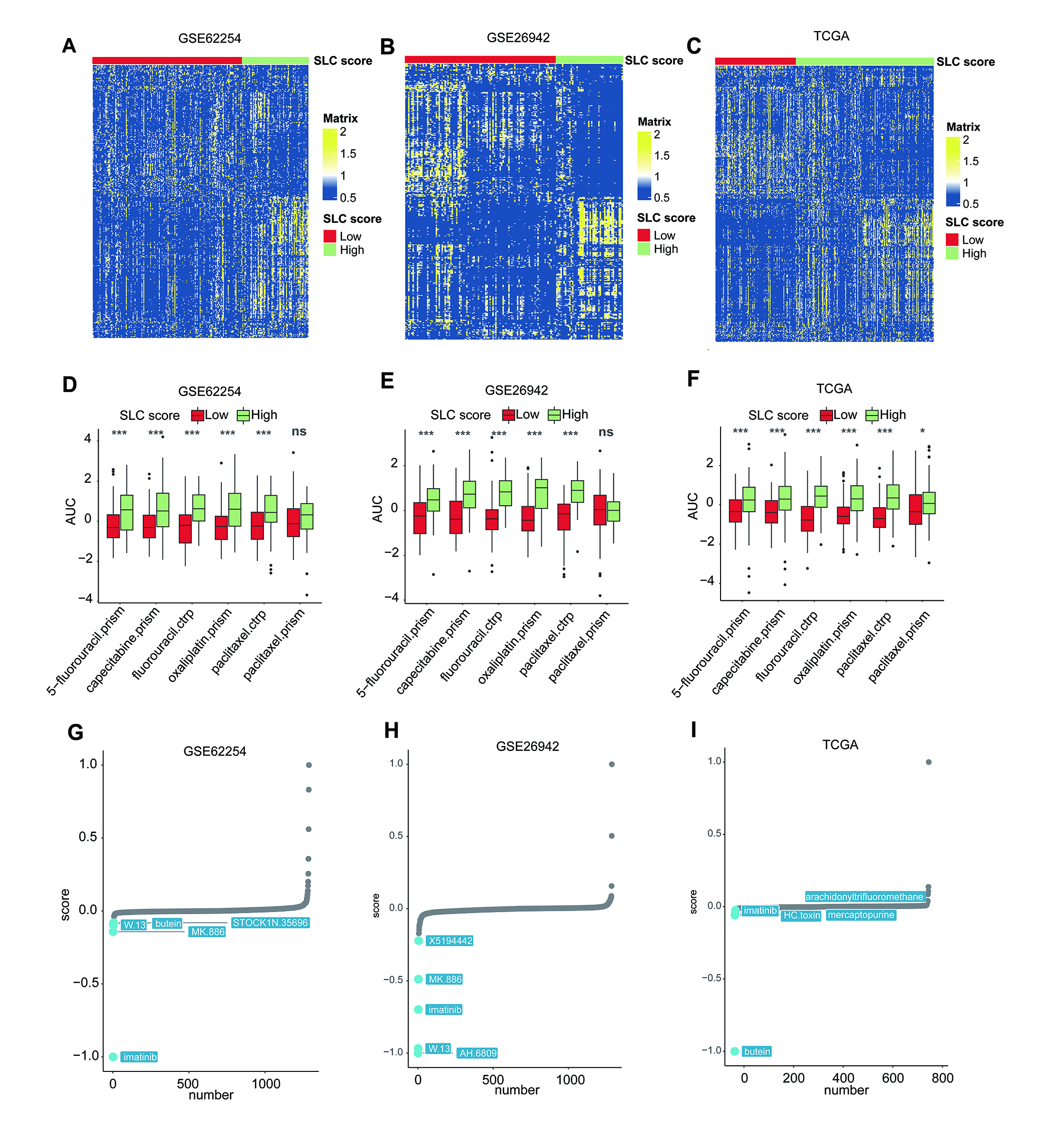

Supplement: Supplementary file 6 — Figure S6 [file JCMM-27-4181-s007.tif]
