# Supplementary material for: Co‐expression pattern of SLC transporter genes associated with the immune landscape and clinical outcomes in gastric cancer
Source: J Cell Mol Med. 2023 Nov 1;27(24):4181–94. doi: 10.1111/jcmm.18003 (PMC10746955; doi:10.1111/jcmm.18003)
Supplement: Supplementary file 13 — Captions Figure and Table S1‐S6 [file JCMM-27-4181-s008.docx]

Captions for Figures S1-S6 and Table S1-S6：

Fig.S1. Workflow of this study.

[Fig.S2.](https://mail.163.com/js6/read/readhtml.jsp?mid=191:1tbivx4G41WB5EXNNgACsI&userType=browser&font=15&color=064977&ignoreMailTracking=1) Impact of different SLC subtypes and SLC gene clusters on clinical outcomes in gastric cancer. (A) The optimal number of clusters was determined using the K-means method and calculating all available evaluation indices using the NbClust function; (B) The optimal number of clusters was determined using the ward.D method and calculating all available evaluation indices using the NbClust function. (C) Forest showed the impact of different SLC subtypes on relapse-free survival (RFS) of patients with gastric cancer in GSE62254 (206 patients), GSE26942 (150 patients), and TCGA-STAD (201 patients) cohorts, respectively. Each small square represents an unadjusted risk ratio, while the horizontal line represents the corresponding 95% confidence interval (CI). The stacked plots were displayed to show the clinical features of different SLC subtypes in the (D) GSE26942 (150 patients) and (E) TCGA-STAD (289 patients) cohorts. Statistical significance was assessed using the chi-square test. **P* < 0.05, ***P*< 0.01, ****P* < 0.001. Multivariate survival analyses for OS (F) and (G) RFS of SLC subtypes, clinical variables, and some molecule types in the GSE62254 cohort (222 patients). Each small square represents an unadjusted risk ratio, while the horizontal line represents the corresponding 95% confidence interval (CI). (H) The correlation matrix showed the correlation between the genes of the SLC-C1 and the genes of the SLC-C2. (I) Forest plot was used to display the impact of different SLC gene clusters on relapse-free survival (RFS) of patients with gastric cancer in GSE62254 (206 patients), GSE26942 (150 patients), and TCGA-STAD (201 patients) cohorts, respectively. Each small square represents an unadjusted risk ratio, while the horizontal line represents the corresponding 95% confidence interval (CI). Abbreviation; SLC-S1, SLC subtype 1; SLC-S2, SLC subtype 2; SLC-C1, SLC gene cluster 1; SLC-C2, SLC gene cluster2; CI, confidence interval; HR, hazard ratios.

Fig.S3. Tumor microenvironment characteristics and activation of biological pathways of different SLC subtypes. (A) Heatmaps exhibited the activation status of biological pathways of different SLC subtypes in the GSE62254 (left, 222 patients), GSE26942 (middle, 150 patients), and TCGA-STAD (right, 289 patients) cohorts. (B) Heatmaps exhibited the tumor microenvironment landscape of different SLC subtypes in the GSE62254 (left, 222 patients), GSE26942 (middle, 150 patients), and TCGA-STAD (right, 289 patients) cohorts. Abbreviation; EMT, epithelial-mesenchymal transition; SLC-S1, SLC subtype 1; SLC-S2, SLC subtype 2.

[Fig.S4](https://mail.163.com/js6/read/readhtml.jsp?mid=191:1tbivx4G41WB5EXNNgACsI&userType=browser&font=15&color=064977&ignoreMailTracking=1). Exploration of the SLC score in gastric cancer. (A) Forest plot of associations between the expression of genes consisting of SLC score and RFS of patients in the GSE62254 cohort (206 patients). Each small square represents an unadjusted risk ratio, while the horizontal line represents the corresponding 95% confidence interval (CI). (B) The principal component analyses demonstrated that patients with SLC-S1 and SLC-S2 subtypes were effectively distinguished based on SLC score in the TCGA-STAD cohort (289 patients). (C) Violin plot (left) of SLC score value in two SLC subtypes, and receiver operating characteristics curve (right) of the SLC score model for the prediction of SLC-S2 subtype in the TCGA-STAD cohort (289 patients). Boxes inside the violins represent 25-75% of values, lines in boxes represent median values, whiskers represent 1.5 interquartile ranges, and black dots represent outliers. Student's t-test was utilized to evaluate statistical significance. **P* < 0.05, ***P*< 0.01, ****P*< 0.001. (D). Correlation matrix showing the correlation between SLC score and the infiltration of TME cells, as well as the correlation between SLC score and activation levels of biological pathways in the TCGA-STAD cohort (289 patients). “pos” represented positive relation; “neg” represented negative relation; “rSeg” represented the absolute value of correlation coefficient; “r” represented correlation coefficient. (E) The TIDE algorithm was used to predict the TIDE values for subgroups with low or high SLC scores in the TCGA-STAD cohort (289 patients) and the results were shown in violin plots. Student's t-test was utilized to evaluate statistical significance. **P*< 0.05, ***P*< 0.01, ****P* < 0.001. Abbreviation; SLC-S1, SLC subtype 1; SLC-S2, SLC subtype 2; CI, confidence interval; HR, hazard ratios.

Fig.S5. Kaplan-Meier curves of OS in the GSE57303 cohort (53 patients) according to the SLC score. The log-rank test was utilized to compare the statistical significance.

Fig.S6. Screening of candidate compounds for gastric patients with high SLC scores. Based on CTRP analysis, heatmaps exhibited predicted AUC values of different compounds corresponding to patients with low or high SLC scores in the (A) GSE62254 (222 patients), (B) GSE26942 (150 patients), and (C) TCGA-STAD (289 patients) cohorts, the lower the AUC value is, the higher the sensitivity is. Based on CTRP analysis, violin plots exhibited predicted AUC values of common chemotherapy drugs corresponding to patients with low or high SLC scores in the (D) GSE62254 (222 patients), (E) GSE26942 (150 patients), and (F) TCGA-STAD (289 patients) cohorts, the lower the AUC value is, the higher the sensitivity is. Student's t-test was utilized to evaluate statistical significance. **P* < 0.05, ***P*< 0.01, ****P* < 0.001. The top five drugs with the lowest CMAP scores corresponding to patients with low or high SLC scores were demonstrated in (G) GSE62254 (222 patients), (H) GSE26942 (150 patients), and (I) TCGA-STAD (289 patients) cohorts, respectively.

Table S1. Basic information of gene expression profiling series.

Table S2. Patients’ basic characteristics.

Table S3. Basic information of SLC family genes included in the current study.

Table S4. Significant mutated genes between SLC-S1 and SLC-S2.

Table S5. Significantly varied methylation genes between SLC-S1 and SLC-S2.

Table S6. Differentially expressed genes in SLC-S2 cluster.
